# How a personalised transportable folding device for seating impacts dysphagia

**DOI:** 10.1007/s00405-019-05657-5

**Published:** 2019-10-04

**Authors:** Virginie Woisard, Mireille Costes, Hélène Colineaux, Benoit Lepage

**Affiliations:** 1 Voice and Deglutition Unit, Otorhinolaryngology Department, Rangueil Larrey University Hospital of Toulouse, 31059 Toulouse Cedex, France; 2 Department of Epidemiology, USMR, 37 Allées Jules Guesde, 31073 Toulouse, France

**Keywords:** Dysphagia, Swallowing disorders, Positioning, Transportable device, Hyoid bone motion

## Abstract

**Purpose:**

A personalised transportable folding device for seating (DATP) on a standard seat was developed by an occupational therapist at the Toulouse University Hospital Centre (patent no. WO 2011121249 A1) based on the hypothesis that the use of a seat to assist with better positioning on any chair during meals modifies the sitting posture and has an impact on cervical statics which increases the amplitude of movements of the axial skeleton (larynx and hyoid bone) and benefits swallowing. The aim of this work is to demonstrate that an improvement in sitting posture with the help of the DATP, through Hyoid bone motion, has an impact on the quality of swallowing in a dysphagic population which benefits from the device in comparison to a dysphagic population which does not benefit from the device after 1 month of care. The secondary endpoints concern the evaluation of the impact on other characteristics of swallowing, posture, the acceptability of the device and the quality of life.

**Methodology:**

This is a randomised comparative clinical trial. The blind was not possible for the patients but the examiner who evaluated the outcome criterion was blinded to the group to which the patient belonged. The outcome criterion was the measurement of the hyoid bone movement during swallowing. The other criteria were collected during the videofluoroscopic examination of swallowing and by use of a questionnaire. Fifty-six (56) patients were included: 30 in the group without device (D−) and 26 in the group with the device (D+). All the patients benefited from a training course on seating. Only the D+ patients participated in this course where the use of the device was explained and the device was then kept for use at home for 1 month.

**Results:**

A significant improvement was noted in the postural criteria before and after use, in favour of a better posture for the two groups (*p* < 0.001) and more hyoid bone motion in the D+ group. The difference was significant in the bivariate analysis for horizontal movement (*p* = 0.04). After adjustment of potential factors of confusion, we noted a significant mean difference for the three distances in the D+ group in comparison to the D− group, of + 0.33 (95% CI [+ 0.17; + 0.48]) for horizontal movement, + 0.22 (95% CI [+ 0.03; + 0.40]) for vertical movement and + 0.37 (95% CI = [+ 0.20; + 0.53]) for horizontal movement. However, the other parameters, and notably the other swallowing markers were not significantly modified by the use of the device.

**Conclusion:**

The personalised transportable folding device for seating developed to reduce dysphagia has an action on hyoid bone motion during swallowing. However, this positive effect on an intermediate outcome criterion of the quality of swallowing was not associated with an improvement in swallowing efficiency in the study population. The diversity of diseases with which the patients in this study were afflicted is a factor to be controlled in future studies with this device.

## Introduction

Many swallowing rehabilitation methods are used in clinical practice: stimulation, dietary adaptations (texture and volume), swallowing exercises, posture and swallowing manoeuvres. Postural adaptations in terms of the positioning of the head are one of the key elements of care regardless of the aetiology [1–4]. To reduce cervical constraints, global positioning adapted to the body is a prerequisite [5]. However, there are very few publications on the effect of the different positions of the body on swallowing. They mainly concern healthy subjects. Rosen et al. [6] analysed the effect of various degrees of body inclination on pharyngeal pressure during swallowing. Yasuhiro et al. [7] noted the effect of excess tension in the anterior cervical musculature on the kinetics of the hyoid bone. Rasley et al. [8] and Byung-Mo et al. [9] reported that the lateral decubitus position decreased aspiration. Publications on the impact of positioning on dysphagia in the presence of postural disorders are even more infrequent. They mostly concern small children [10] and disabled adults [11].

The most obvious method of action is the adaptation of the seat according to the morphology and the deficiencies. When the seat is a vehicle for a disabled person, the postural adaptation method is known [12]. For subjects who use a standard seat, a chair or different seats according to the place and the time, there is no satisfactory equipment.

A personalised transportable folding device for seating (DATP) placed on a standard seat is a solution that could find relevance in the home, in adult disability care homes or in elderly care facilities. It is in this context that a device was developed by an occupational therapist in our deprtment and patented (WO 2011121249 A1). Prior to the creation of this device, training sessions were carried out. However, the patients did not benefit from technical assistance to implement them at home [13]. The DATP was used to support a patient and/or caregiver training programme to help them with adapted positioning. It could then be kept by the patient if the capacities for posture control remained inadequate despite training.

This work is based on the hypothesis that assistance with better positioning by using a seat on any standard chair during meals modifies the sitting posture with an effect on cervical statics and provides greater amplitude in axial skeleton (larynx and hyoid bone) movements which helps with swallowing and changing postures.

Our aim is to demonstrate that an improvement in sitting posture with a sitting device has an impact on the quality of swallowing.

The main endpoint of this work is to compare hyoid bone motion during swallowing in a dysphagic population which benefits from the device in comparison to a dysphagic population which does not benefit from the device after 1 month of care.

The secondary endpoints concern the evaluation of the impact on other characteristics of swallowing, posture, the acceptability of the device and the quality of life.

## Materials and methods

### Study design

This is a prospective, monocentric controlled study with the design of a superiority randomised controlled clinical trial (1:1) in two parallel groups. The blind is not possible for patients,s but the examiner who evaluated the outcome criterion was blinded to the group to which the patients belonged.

### Population

The patients in this study were required to have a sitting abnormality determined by the seated postural control measure (SPCM, [14, 15]) and dysphagia determined by the Deglutition Handicap Index (DHI, [16–18]). They were exhaustively and successively recruited over a period of 3 years (from 17/2/2015 to 14/03/2017) when they were seen for a swallowing assessment at the voice and deglutition unit. The inclusion criteria were the following: age > 18 years; DHI score above 11, score of more than 0 for at least 1 of the following 3 items on the SPCM: pelvic retroversion, obliquity and rotation; the preservation of autonomy compatible with the use of various seats including standard seats; absence of Spinal rigidity or irreducible hips (spondylarthritis, osteosynthesis rod); and chronic dysphagia.

### Procedure

#### Device

The device that was tested is a sitting aid prototype. It targets a population with postural disorder and dysphagia that has the capacity to ambulate which justifies a device that can be easily and rapidly installed on several types of seats.

It concerns a transportable folding device for maintaining a seated posture, which includes: a bottom lining suitable for a seat, where the said bottom lining includes a rigid seat and a backrest, with the seat and backrest connected to each other in alignment by a link that forms a hinge; at least one orthopaedic support element suitable for the bottom lining and an attachment to fit the orthopaedic support element to the bottom lining by adjusting the position.

This device was used as technical support in a training programme to improve the sitting position.

All the patients benefited from a training session including an evaluation of their needs, information on the impact of head positioning on swallowing, on how to facilitate an adapted position of the head through global positioning of the body with practice sessions using occupational therapy cushions or the DATP according to the randomisation.

#### Study procedure

During a visit to the unit for a swallowing assessment, the patients who met the inclusion criteria were scheduled for an inclusion visit and the first data were collected: age, gender, weight and BMI, dysphagia history, dysphagia aetiology, DHI, the treatment implemented for dysphagia (notably physical therapy and/or speech therapy) and 3 items of the SPCM. This corresponded with the pre-inclusion visit (T-1 month).

The inclusion visit corresponds to T0. After verification of the inclusion criteria, the patients were randomly distributed into the arm with the device (D+) or without the device (D−). All the patients benefited from the same assessment:

- The complement to the SPCM items by the occupational therapist.
- A videofluoroscopy of swallowing.
- The Functional Oral Intake Scale (FOIS [19]) and the SF36 [20] questionnaires.

The two patient groups were then interviewed to establish a diagnosis for educational purposes and the objectives of the training session were thereby specified and personalised.

The D+ group was then taken in charge to determine the characteristics of the device required for the order so that they could have them during the training session.

All the patients were re-invited for the therapeutic training session run by the occupational therapist 1 month after (T + 1 month). This deadline was justified by the deadline for manufacture of the device by the company for the patients in the D+ group. The instruction for patients at the end of this training session was to put the personalised instructions into practice by using the device only for the D+ group.

**Fig. 1:**
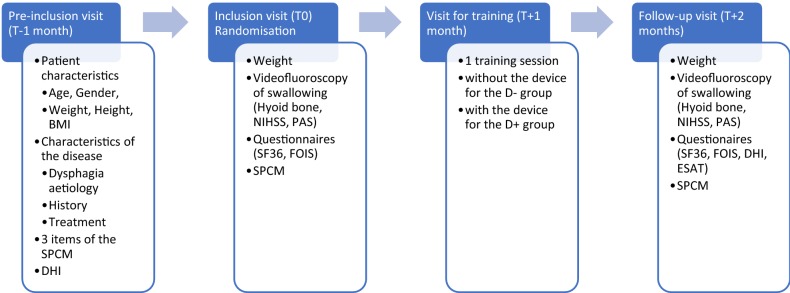
Study procedure and summary of the data collected

At the control visit 1 month after at T + 2 months, all the outcome criteria at T0 were collected in addition to the DHI which was collected at T-1 month and the device satisfaction questionnaire for the patients in the D+ group (Fig. 1).

### Outcome criteria

The outcome criteria were measured at pre-inclusion (T-1), during inclusion (T0) and in 2 months of follow-up (T2). The effectiveness of the device was evaluated by the improvement in swallowing based on the laryngeal rise criterion, which is recognized as a reliable indication of laryngeal closure during swallowing [21].

#### Main outcome criterion

The main outcome criterion was the measurement of laryngeal movement during swallowing, which is one of the parameters that describe the biomechanics of swallowing [4]. For this, hyoid bone motion is the most reliable marker and is measurable from videofluoroscopy recordings of swallowing (VFS), the gold standard of clinical exploration of dysphagia. We used the technique described by Paik et al. [22].

The VFS was carried out as follows for all the patients:

- Positioning of the patient side-on seated on a standard chair.
- A 10c Euro coin stuck to the skin in the cervical region under the chin served as a reference for measurement.
- A bolus of 5 ml barium sulphate diluted to 35% W/V repeated five times.

The maximum hyoid bone excursion was calculated after location of the anterior and superior edge of the hyoid bone. The movement in cm was measured on a vertical axis and on a horizontal axis with the following marking:

- *Y* axis defined by a straight line passing through the following two points:
- the inferior and anterior edge of C4 = point 0,
- the inferior and anterior edge of C2,
- *X* axis strictly perpendicular to the *y* axis at point 0 (inferior and anterior edge of C4),

All the swallowing could not be exploited for the main measurement due to errors in centring and errors in synchronisation between the start of swallowing and recording.

At T0 and T2, the swallowing measurements were taken based on three videofluoroscopic recordings that provided conditions for satisfactory marking and selected in a standardised manner from among all the videofluoroscopic recordings of swallowing.

The outcome criteria were the mean of these three measurements for three dimensions: vertical, horizontal and total movements (hypotenuse), after evaluation of the measurement concordance. Technically speaking, the video recordings were manual [23].

These criteria were measured several times. Therefore, the measurement concordance was evaluated by an intra-class coefficient of correlation (ICC). This was satisfactory for the measurements of hyoid bone motion: the ICC for the mean of the measurements for horizontal movement was 0.85 at T0 and 0.90 at T2, for vertical movement 0.89 at T0 and 0.88 at T2 and for global movement (hypotenuse) 0.87 at T0 and 0.88 at T2.

It was also satisfactory for the NIHSS (0.96 at T0 and 0.96 at T2). However, it was marginal for the PAS measurement (0.66 at T0 and 0.69 at T2).

#### Secondary outcome criteria

The other criteria made it possible to complete the analysis of the impact on swallowing, confirm the impact on posture and verify the acceptability of the device and the quality of life.

- The other outcome criteria collected during the videofluoroscopic examination were:
  - The NIH Swallowing Safety Scale (NIH-SSS) which estimates the global swallowing efficacy [24] for which the score is comprised between 0 (normal) and 10 + (very altered).
  - The Penetration Aspiration Scale (PAS) which evaluates aspiration with an ordinal scale at 8 levels: 1 (no aspiration and 8 (severe aspiration) [25].
- The other swallowing and feeding markers were:
  - FOIS [19]: ordinal scale at 7 levels that reflects the patient’s feeding situation (1—no oral feeding, 7—normal oral feeding).
  - The Deglutition Handicap Index (DHI, [16]) which measures the quality of life related to dysphagia comprised of three areas rated from 0 to 40, present a total between 0 and 120.
- For posture, anomalies of the seated position and the impact of the device were evaluated,
  - A seated postural control measure validated for subjects seated in wheelchairs was used. The SPCM includes 21 items rated on the equivalent of an ordinal scale at 7 levels. The scores can be grouped together.
  - For the acceptability of the device, satisfaction with a technical aid was evaluated with a Quebec User Evaluation of Satisfaction with Assistive Technology (QUEST [26]) questionnaire which includes 12 items, 8 of which concern the degree of satisfaction with the technology, 4 concern the related services. The sources of dissatisfaction were also identified. The items were rated on an ordinal scale at 5 levels, ranging from “not at all satisfied” to “very satisfied”. Total scores were obtained by calculating the mean of the scores. The sources of dissatisfaction were reported qualitatively.
- For the quality of life the SF36 [20] questionnaire was used. Each question is standardised on a scale of 0–100.

### Sample size

Paik et al. [22] showed that patients with stroke versus controls had respectively a horizontal excursion of hyoid bone of 1.1 cm ± 0.2 cm and 1.5 cm ± 0.1 cm. Assuming standard errors of 0.3 cm, 22 patients in each group (total *N* = 44) were required to detect differences greater than 0.3 cm, at an *α* level of 5% and power of 90%. Assuming a 30% loss of the patients to follow-up, the sample size was increased to 32 in each group (total *N* = 64).

### Randomisation

The random allocation sequence was obtained using the *ralloc* package for Stata 12 SE, in blocks of sizes that vary between 2, 4 and 6

The evaluation of the outcome criteria in videofluoroscopy was blinded. The video recording of each swallowing sequence related to a bolus was prepared so that it could be identified by a random number independent of the evaluation time (T0 or T2) and the patient’s identity. The practitioner then analysed the recordings in the order of this random numbering. The intervention was open for the patients (a blind is impossible with this device) and for the evaluation of the other outcome criteria.

### Statistical analysis

All the variables measured at inclusion or pre-inclusion (characteristics of the patient and the expectation) were described for the total population. The initial comparability of the groups was verified by the description of these same variables by randomisation group (with and without the positioning device).

The main and secondary outcome criteria were compared at the last visit (*t* + 2 months) with a Student test or a Mann–Whitney–Wilcoxon test. Then, for the main outcome criteria, three linear regressions were performed to model the movement (global, horizontal or vertical) according to the intervention group, after adjustment of the factors of confusion identified in principle: the quality of initial swallowing (global, horizontal or vertical movement at T0), malnutrition measured by weight, severity of the postural effect (measured by the total SPCM score at T), the prognosis through age and the type of disease (degenerative or non-degenerative), and the associated treatment (speech and/or physical therapy).

The analyses were carried out with Stata Version 14.2 (StataCorp LP, College Station, TX, USA).

### Ethical statement

This study received the approval of the committee for the protection of individuals CCP SOOM 2 (ethical committee) on 05/05/2014 under no. 2-14-08.

The RCB number (HPS Study) is 2013-A01351-44.

## Results

### Population

**Fig. 2:**
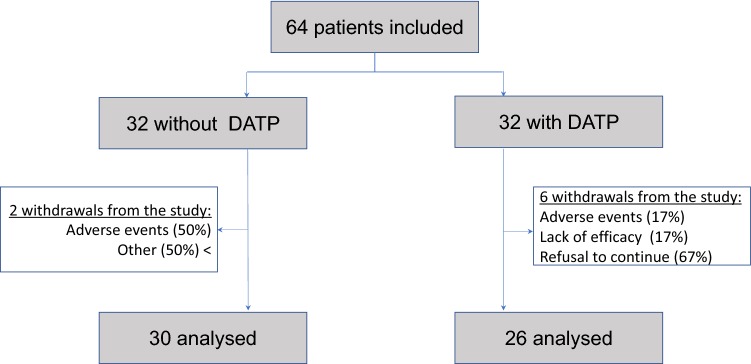
Flow chart

Of the 64 patients included in the study, 32 were randomised in the group with DPAT and 32 in the group without DPAT. Eight withdrew from the study after randomisation: two in the “D+” group and six in the “D−” group: two due to the occurrence of adverse effects, one due to a rapid worsening of dysphagia, four due to a decision by the patient to withdraw = one refused to continue and 1 was lost to follow-up (Fig. 2). The adverse effects had no direct relationship with the study (context of depression and a road accident). The number of subjects analysed was therefore 56.

The initial characteristics of the population included are provided in Table 1, globally and by group. The population was mainly male (35 men), i.e. 37.5% women and 62.5% men. The mean age was 61.5 years (range 30–82, SD 11.8).

**Table 1:** Outcome criteria per group

	No DATP (*N* = 30)		DATP *N* = 26)			Mean difference^a^ DATP versus no DATP			
	*m*	SD	*m*	SD	*p*	*m*	(CI 95%)		*p*
Swallowing—feeding									
Horizontal movement (cm)	0.68	0.39	0.93	0.46	0.04^S^	0.33	(0.17; 0.48)		< 0.01
Vertical movement (cm)	1.27	0.47	1.39	0.58	0.40^S^	0.22	(0.03; 0.40)		0.03
Global movement (cm)	1.51	0.46	1.76	0.51	0.06^S^	0.37	(0.20; 0.53)		< 0.01
NIHSS	1.3	1.4	1.3	1.6	0.75^W^				
PAS	1.7	1.3	1.9	1.9	0.53^W^				
FOIS	6.0	0.9	5.8	1.1	0.72^W^				
DHI									
Physical	13.4	6.6	13.5	6.5	0.94^S^				
Functional	10.5	6.6	12.5	7.3	0.30^S^				
Emotional	8.5	7.9	11.7	9.1	0.27^W^				
Total	32.4	17.5	37.8	22.4	0.31^S^				
Posture									
SPCM by plane									
Sagittal	9.1	2.9	1.9	2.0	< 0.01^W^				
Frontal	2.5	1.4	0.5	0.6	< 0.01^W^				
Horizontal	3.5	1.6	0.8	0.9	< 0.01^W^				
Total	15.1	3.8	3.2	2.2	< 0.01^W^				
SPCM by segment									
Pelvis	3.1	1.1	0.4	0.6	< 0.01^W^				
LL	6.5	2.7	1.2	1.3	< 0.01^W^				
Torso	4.1	1.5	1.0	1.0	< 0.01^W^				
Head	1.4	0.9	0.6	0.6	< 0.01^W^				
Segment total	18.2	4.5	3.7	2.7	< 0.01^W^				
Quality of life									
RAND SF36									
Physical functioning PF	60.8	33.2	53.3	28.3	0.26^W^				
Physical role PR	55.0	44.2	50.0	41.8	0.67^S^				
Bodily pain BP	60.8	24.0	60.4	27.9	0.95^S^				
General health GH	43.7	19.9	48.3	22.4	0.32^W^				
Vitality VT	43.3	18.4	48.5	21.0	0.74^W^				
Social function SF	66.7	22.1	61.0	27.1	0.37^W^				
Emotional role ER	48.9	45.3	48.7	48.3	0.99^S^				
Mental health MH	59.1	18.0	60.2	22.0	0.84^S^				

*S* Student Test, *W* Mann–Whitney–Wilcoxon Test, *p p* value

^a^Adjusted analysis of movement (horizontal/vertical/or global), weight, SPCM score, age, the presence of a degenerative disease, physiotherapy/speech therapy

Dysphagia was considered to be degenerative, i.e., with a tendency to worsen over time, for 43% (*n* = 24) of the patients and included the following neurological diseases: extrapyramidal syndromes and Huntington’s disease (*n* = 12) neuromuscular disease and myopathy such as ALS, Steinert’s disease (*n* = 9) Scleroderma (*n* = 3). The other diseases (57.1%; *n* = 32) were brain lesions (CVA, head trauma) (*n* = 3), peripheral neuropathy and bilateral cranial nerve palsy (*n* = 7), head and neck cancer (*n* = 8), respiratory or digestive disease (*n* = 7), laryngeal or cervical disease (*n* = 7). It should be noted that 11 (19.6%) patients had no disease associated with the cause of the dysphagia, 15 had an associated disease (26.8%), 30 patients had more than one associated disease. The history of the disorder was between 3 months and 57 months with a mean of 10.1 months (SD 11.2). No other care was proposed in addition to postural training during the study period for 56.4% of the patients. 12.7% continued physical therapy alone, 16.4% speech therapy alone and 14.6% both.

No major difference was noted between the two groups on the initial patient profile, including on the outcome criteria that were used in this study.

### Change in posture and swallowing

After 2 months of follow-up, the SPCM scores showed a significant improvement in the postural criteria in favour of a better posture in the groups with and without DATP (*p* < 0.001 in bivariate analysis) (Table 1).

**Fig. 3:**
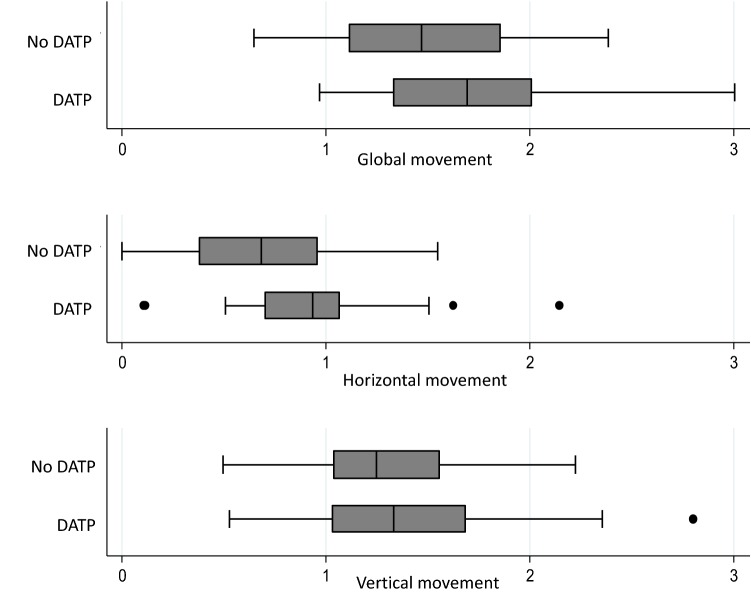
Movement at T2 per group

For Hyoid bone movement, we observed more movement in the DATP group than in the group without DATP (Fig. 3):

- the difference was significant in the bivariate analysis for horizontal movement (*p* = 0.04),
- after adjustment on the potential factors of confusion, we observed a significant mean difference for the 3 distances in the group with DATP in comparison to the group without DATP: + 0.33 (95% CI = [+ 0.17; + 0.48]) for horizontal movement, + 0.22 (95% CI = [+ 0.03; + 0.40]) for vertical movement and + 0.37 (95% CI = [+ 0.20; + 0.53]) for global movement (Table 2).

**Table 2:** Initial characteristics of the population

	All together (*n* = 56)		No DATP (*N* = 30)		DATP (*N* = 26)	
	*m*	SD	*m*	SD	*m*	SD
Initial characteristics						
Age	61.5	11.8	59.0	13.4	64.3	9.1
Gender						
Female, *n* (%)	21	(37.5)	11	(36.7)	10	(38.5)
Male, n (%)	35	(62.5)	19	(63.3)	16	(61.5)
Weight	68.2	13.2	66.8	13.6	70.0	12.8
Type of disease						
Non-degenerative, *n* (%)	30	(53.6)	16	(53.3)	14	(53.8)
Degenerative, *n* (%)	26	(46.4)	14	(46.7)	12	(46.2)
Physiotherapy and/or speech therapy						
No, *n* (%)	31	(55.4)	16	53.3	15	57.7
Yes, *n* (%)	25	(44.6)	14	46.7	11	42.3
Swallowing—feeding						
Horizontal movement (T0)	0.76	0.37	0.78	0.40	0.73	0.34
Vertical movement (T0)	1.21	0.54	1.29	0.45	1.11	0.63
Global movement (T0)	1.51	0.49	1.58	0.44	1.43	0.53
NIHSS (T0)	1.4	1.6	1.3	1.5	1.4	1.8
PAS (T0)	1.8	1.6	1.9	1.4	2.2	2.1
FOIS (T0)	5.8	1.0	5.9	0.9	5.7	1.0
DHI (T-1)						
Physical	15.1	6.0	14.4	5.3	16.0	6.7
Functional	12.4	7.5	11.9	6.6	12.9	8.6
Emotional	11.0	8.2	11.1	6.9	10.8	9.5
Total	38.5	19.1	37.4	16.3	39.7	22.2
Posture						
SPCM by plane (T0)						
Sagittal T0	9.8	3.5	8.7	3.8	11.0	3.5
Frontal T0	2.6	1.6	2.9	1.8	2.3	1.4
Horizontal T0	2.9	1.7	3.0	2.0	2.9	1.3
Total T0	15.3	3.9	14.6	4.2	15.3	3.6
SPCM by segment (T0)						
Pelvis T0	2.7	1.1	2.7	1.1	2.7	1.3
LL T0	7.1	2.9	6.3	2.8	8.2	2.6
Torso T0	4.1	1.5	4.2	1.4	4.0	1.5
Head T0	1.3	1.0	1.4	1.1	1.2	0.8
Segment total T0	18.0	4.7	17.3	4.9	18.8	4.4
Quality of life						
RAND SF36 (T0)						
Physical functioning PF	59.0	30.9	62.2	32.1	55.4	29.7
Physical role PR	52.7	41.7	49.2	45.2	56.7	37.8
Bodily pain BP	57.1	23.3	61.8	20.7	51.5	25.5
General health GH	45.8	18.5	47.0	18.7	44.4	18.6
Vitality VT	43.2	18.4	43.7	16.2	42.7	20.8
Social function SF	59.2	22.8	60.8	22.4	57.2	23.5
Emotional role ER	53.6	41.0	50.0	43.5	57.7	38.4
Mental health MH	57.1	17.5	56.3	15.8	58.5	19.6

*m* mean, *SD* standard deviation, *n* number of subjects, % percentage

However, the other parameters, and notably the other swallowing markers (NIHSS, PAS, FOIS, DHI) were not significantly modified by the use of the device. In addition, no significant improvement in the different dimensions of quality of life were noted (SF36).

### Acceptability

**Fig. 4:**
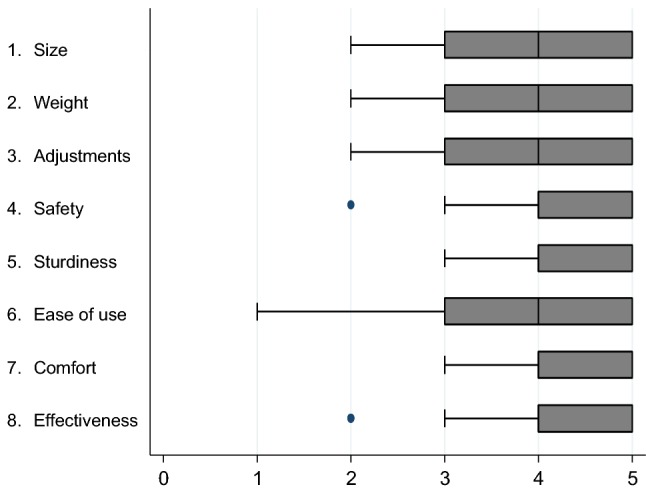
Device acceptability scores

The acceptability of the device was very good with a mean total score of 4.0 out of 5 (SD 0.7). All the dimensions evaluated had a mean score of more than 3.5 (Fig. 4). The advantages of the device most often cited by the users were: the ease of use (27% of the users consider this the main advantage), the size (27%) and the capacity to be adjusted (15%).

## Discussion

Improvement in posture is an important area of care for patients with chronic dysphagia. It is all the more important because the aetiology of the disorder is associated with a body statics disorder as is the case in neurological diseases.

The results of this work confirm that a device such as the DATP improves sitting body statics and show the impact on the dynamics of the hyoid bone during swallowing. They provide arguments for a cause and effect relationship between an improvement in posture and a gain in the amplitude of movements of the hyoid bone in a population of dysphagic patients. However, they provide no indication of improvement in dysphagia. Is this related to the difficulty documenting the results, to the diversity of the study population, the low benefits in terms of mobilisation of the Hyoid bone?

The maximum measure of Hyoid bone amplitude found in our work is consistent with the data in the literature. In the study that serves as our reference, the mean amplitude of horizontal movement in the hyoid bone was 1.5 + -0.1 cm for control subjects, 1.1 ± 0.2 for CVA patients and 0.4 ± 0.1 for inflammatory myopathy patients while it was 0.76 ± 0.37 in all diseases together in our study. For vertical movement, it was respectively 1.3 ± 0.3 for the controls, 1.2 ± 0.1 for the CVA patients and 0.8 ± 0.3 for the inflammatory myopathy patients [22]. It was 1.21 ± 0.54 cm in our study. As was the case in the study by SIA et al. [27], the reproduction of the intra-rater measure was satisfactory in favour of the reliability of the repetition of the measure but also indicates little difference in the 2 to 5 cases of swallowing evaluated.

The effect was at a maximum on horizontal movement (+ 0.33, 95% CI = [+ 0.17; + 0.48]). We found no publication that enabled us to compare the results. However, in relation to the impact of the changes from the upright seated position and the lateral decubitus position, the difference appears to be inferior: 2 mm in bivariate analyses in our study and 3 mm in the study by Byung-Mo Oh et al. [9].

The impact on the axis change measurement in the vertebral column induced by postural correction could influence the results. Sia et al. [27] showed that the rotation of the measurement axis impacted the measurement of horizontal movement. That is why the measurements were taken for all the subjects with a rotation that brings axis Y to the vertical line. Despite this the work conducted by Sia et al. [27] quantified the error in measurement of hyoid bone motion by 2.48–3.06 mm. The horizontal axis movement in our study was slightly more than 3.33.

Finally, the latest works on hyoid bone kinematics put forward as more pertinent [28] the measurements of the hyoid bone motion peak. Semi-automatic measurement techniques are being developed [29] to encourage the use of these parameters in the future.

The absence of impact on the swallowing parameters is probably related to the inadequacy of the effect on swallowing efficiency. In fact, the only positive result of our study concerns an intermediate outcome criterion. We have no data concerning the minimum threshold of hyoid bone motion that makes it possible to judge swallowing efficiency. Molfenter and Steele [30] were unable to show a significant correlation between Hyoid bone motion and the occurrence of aspiration. Only one tendency between the degree of vertical movement expressed in % of C2–C4 distance (mean difference 1% *p* = 0.059) was found in a study population of 13 patients who presented with aspiration versus 29 patients who presented no aspiration. In our study, the poor reproducibility of the PAS measurement on the patient recordings does not make it possible to conclude on this question. This raises the point concerning the sensitivity of the measurement scales available to evaluate swallowing.

Finally, as the studies that have been conducted attest, the reduction in hyoid bone motion is dependent on the pathological context. Wide variations are possible as the study by Paik [22] shows. It is possible that our study gives results on the other swallowing parameters in a more homogeneous population in terms of dysphagia and postural disorders. That is why we plan to continue to validate the DATP in a homogeneous population of patients with Parkinson’s disease by providing a longer period of follow-up that enables the inclusion of clinical outcome criteria on the complications of dysphagia such as the change in nutritional status and the occurrence of pulmonary complications.

## Conclusion

The personalised transportable folding device for seating developed to reduce dysphagia has an action on hyoid bone motion during swallowing. However, this positive effect on an intermediate outcome criterion of the quality of swallowing was not associated with an improvement in swallowing efficiency in the study population. The diversity of diseases with which the patients in this study were afflicted is a factor to be controlled in future studies with this device.
